# Manganese-Induced Parkinsonism and Parkinson’s Disease: Shared and Distinguishable Features

**DOI:** 10.3390/ijerph120707519

**Published:** 2015-07-06

**Authors:** Gunnar F. Kwakye, Monica M.B. Paoliello, Somshuvra Mukhopadhyay, Aaron B. Bowman, Michael Aschner

**Affiliations:** 1 Neuroscience Department, Oberlin College, Oberlin, OH 44074, USA; E-Mail: gkwakye@oberlin.edu; 2 Graduate Program in Public Health, Department of Pathology, Clinical and Toxicological Analysis, Center of Health Science, State University of Londrina, Parana 10011, Brazil; E-Mail: monibas2@gmail.com; 3 Division of Pharmacology and Toxicology, College of Pharmacy, Institute for Cellular & Molecular Biology, and Institute for Neuroscience, The University of Texas at Austin, Austin, TX 78712, USA; E-Mail: som@austin.utexas.edu; 4 Department of Neurology and Pediatrics, Vanderbilt University, Nashville, TN 37240, USA; E-Mail: aaron.bowman@vanderbilt.edu; 5 Department of Molecular Pharmacology, Albert Einstein College of Medicine, Bronx, NY 10461, USA

**Keywords:** manganese-induced parkinsonism, manganese neurotoxicity, Parkinson’s disease (PD), manganism, neurodegenerative diseases

## Abstract

Manganese (Mn) is an essential trace element necessary for physiological processes that support development, growth and neuronal function. Secondary to elevated exposure or decreased excretion, Mn accumulates in the basal ganglia region of the brain and may cause a parkinsonian-like syndrome, referred to as manganism. The present review discusses the advances made in understanding the essentiality and neurotoxicity of Mn. We review occupational Mn-induced parkinsonism and the dynamic modes of Mn transport in biological systems, as well as the detection and pharmacokinetic modeling of Mn trafficking. In addition, we review some of the shared similarities, pathologic and clinical distinctions between Mn-induced parkinsonism and Parkinson’s disease. Where possible, we review the influence of Mn toxicity on dopamine, gamma aminobutyric acid (GABA), and glutamate neurotransmitter levels and function. We conclude with a survey of the preventive and treatment strategies for manganism and idiopathic Parkinson’s disease (PD).

## 1. Mn Essentiality and Uses

Mn is an essential and abundant micronutrient required for normal development and growth [1,2]. It is present at low concentrations in legumes, pineapples, beans, nuts, tea, and grains [3,4]. Importantly, Mn is required for physiological blood sugar regulation and bone formation, immune response, reproduction, as well as lipid, protein and carbohydrate metabolism [5,6,7,8,9]. Mn functions as a cofactor for enzymes such as glutamine synthetase, pyruvate decarboxylase, serine/threonine protein phosphatase I, Mn-superoxide dismutase (Mn-SOD) and arginase, which are required for neurotransmitter synthesis and metabolism, as well as for neuronal and glial function [6,7,9]. Mn is also necessary for cell adhesion and the induction of stellate process formation in cultured astrocytes [10]. Mn is the fourth most widely used heavy metal in the world and exists in 11 oxidation states [2] with Mn^2+^ and Mn^3+^ being the most common in biological systems [11]. The multiple chelate (aspartate, succinate) and salt (sulfate, gluconate) forms of Mn enables for its versatile use in the production of dry cell batteries, the fuel additive methylcyclopentadienyl manganese tricarbonyl (MMT), fungicides (e.g., maneb and mancozeb), paint and adhesives. Other uses of Mn include: (i) iron and steel production; (ii) production of potassium permanganate used as a disinfectant; (iii) oxidant in the production of hydroquinone; (iv) manufacture of glass and ceramics; (v) matches and fireworks; (vi) textile bleaching; (vii) oxidizing agent for electrode coating in welding rods; (viii) leather tanning; and (ix) decolorizing glass [12,13,14]. While uncommon, Mn deficiency can contribute to bone deformities, feebleness, and prolonged susceptibility to seizures, birth defects and diminished reproduction [15,16].

## 2. Mn Toxicity

Despite its essentiality, excessive and prolonged inhalation of Mn particulates in mining, welding and industries results in its accumulation in selected brain regions that causes central nervous system (CNS) dysfunctions and an extrapyramidal motor disorder, referred to as manganism [3,17,18,19]. Prolonged and chronic occupational exposure to Mn (>1 mg/m^3^) represents a risk factor Parkinson’s disease (PD) [20]. Mn uptake and efflux in the brain are stringently regulated under physiological conditions to obtain approximately 1–2 µg/g in dry weight [21]. After elevated exposures, Mn distribution and levels are heterogeneous with the highest level of accumulation observed in the globus pallidus [21,22]. More recent reports suggest that aspects of the disease may also occur in individuals exposed to Mn from environmental sources [23]. In addition, Mn-induced parkinsonism may occur in patients with chronic liver failure who fail to adequately excrete Mn in bile [24] and in individuals receiving total parenteral nutrition [24,25,26,27,28] without exposure to elevated Mn. Further, humans and rats with chronic iron deficiency accumulate Mn in the basal ganglia [29,30] and this has been postulated to be due to the competition of Fe and Mn for shared metal transporters [31]. Finally, patients subjected to microdialysis due to chronic renal failure may develop Mn-induced parkinsonism in the absence of exogenous Mn particulate exposures [32,33].

## 3. Occupational Mn-Induced Parkinsonism

Occupational Mn-induced parkinsonism may occur after prolonged inhalation of Mn fumes and dusts [25]. Individual Mn particles in fumes can be less than 0.01 µm in size [34], but agglomeration may result in particulate aggregates that approach 1 µm in diameter [35]. Indeed, greater than 90% of the Mn containing welding aerosols can be deposited in the lungs due to their small particulate size of <10 µm [36]. Long and colleagues have shown that aerosols from smelting operations may contain substantial levels of biologically relevant non-respirable Mn particles of sizes between 10–100 µm that may permeate the gastrointestinal tract and cause Mn absorption at similar rates as observed in oral Mn intake [36]. Importantly, The American Conference of Governmental Industrial Hygienists (ACGIH) recently recommended a 10-fold reduction in the current threshold limit value time-weighted average (TLV-TWA) for inhaled Mn particles measured over an eight-hour shift from 0.2 mg/m^3^ to 0.02 mg/m^3^ [37].

Despite the unknown prevalence of occupationally mediated Mn-induced parkinsonism worldwide, Mn-induced parkinsonism ranks among the 10 leading occupational disorders in China [38] with an estimated rate of 0.5%–2% in silico- and ferromanganese production plant workers in China [39]. A geographic information system studies in the US has shown a correlation between regions of the US with high industrial Mn emissions and the increased incidence of Mn-induced parkinsonian symptoms and associated mortality [40]. Furthermore, the legal litigation costs relating to occupational manganism in the US amongst 750,000 welders has been estimated to be in the billions [25].

## 4. Modes of Mn Transport

Intracellular levels of Mn are maintained within a narrow physiologic range due to the opposing actions of influx and efflux transporters. Studies on Mn transport in mammalian systems have largely focused on influx mechanisms, and outstanding reviews are available on this subject [41]. To summarize here, Mn is transported into cells by a number of transporters, including divalent metal transporter 1 (DMT1) [42,43], the solute carrier ZIP-8 [44], transferrin receptor [45,46], and calcium channels [47], to name a few. None of these influx transporters appear to be specific for Mn (*i.e.*, they transport other metals in addition to Mn). Studies in yeast indicate that cellular levels of Mn influx transporters are homeostatically regulated in a manner that reduces influx during exposure to elevated Mn and increases influx during Mn deficiency [48,49]. Similar homeostatic regulation of Mn influx was also reported in *C. elegans* [50]. However, in mammalian systems, convincing evidence of homeostatic control of Mn influx has not yet been obtained. More recently, the role of efflux in maintaining cellular Mn levels has begun to be appreciated. To date, three separate transporters/pumps have been found to be capable of mediating Mn efflux: The solute carriers, ferroportin and SLC30A10, and the P-type ATPase secretory pathway Ca-ATPase 1 (SPCA1) [51,52,53,54,55,56]. While there is clear evidence supporting the ability of both ferroportin and SPCA1 to mediate Mn efflux and detoxification in cell culture [51,53,55,56], their roles in mediating Mn detoxification at the whole organism level remains to be clarified. In contrast, SLC30A10 appears to play a fundamental role in maintaining cellular Mn levels and protecting against Mn toxicity at the whole organism level. Recent genetic studies have shown that homozygous mutations in SLC30A10 lead to the onset of a familial Mn-induced parkinsonism [57,58,59,60]. In this disease, patients exhibit 10-20-fold increase in blood Mn, have Mn deposition in the brain and liver, and exhibit parkinsonian symptoms [57,58,59,60]. Importantly, the increased Mn retention in the disease occurs in the absence of exposure to elevated Mn, indicating that loss-of-function mutations in SLC30A10 are associated with Mn retention [58,59,60]. Mechanistic studies performed in 2014 revealed that the wild-type SLC30A10 protein traffics to the cell surface and mediates Mn efflux [52]. In contrast, disease-causing mutations trap the transporter in the endoplasmic reticulum, and these mutants fail to mediate Mn efflux [52]. Mutations in SLC30A10 are the only genetic factor known to be associated with a familial Mn-induced parkinsonian syndrome, and these results highlight the importance of SLC30A10 and efflux in regulating Mn levels at the cellular and organismal level.

## 5. Detection and Pharmacokinetic Modeling of Mn Trafficking

The paramagnetic nature of Mn allows for detection and examination of Mn trafficking dynamics and pharmacokinetics using sensitive and non-invasive methods including positron emission tomography (PET), single-photon emission computed tomography (SPECT) and magnetic resonance imaging (MRI) [61,62,63,64]. Other analytical methods including atomic absorption spectroscopy (AAS), atomic emission spectroscopy (AES), inductively coupled plasma-atomic emission spectrometry (ICP-AES), mass spectrometry (ICP-MS), neutron activation analysis, X-ray fluorometry, spectrophotometry, and radioactive trace assay and cellular fura-2 manganese extraction assay (CFMEA) are used to measure Mn levels in biological specimens [65,66,67]. Chronic high- (>1 mg/m^3^) and low-levels (0.5–1.0 mg/m^3^) of Mn inhalation exposures in the workplace have been reported to result in Mn accumulation in the brain and cause Mn-induced parkinsonism and subtle subclinical changes in the general population respectively [68,69,70,71,72]. The concerns that chronic low-level Mn inhalation exposure may be associated with subtle, subclinical neurological changes have led to the development of pharmacokinetic data sets and physiologically based pharmacokinetic models (PBPK) in adult monkeys and rats [73,74], PBPK models of gestation and lactation in rats [75,76], and a PBPK model in humans [77] to predict inhalation exposure conditions that result in increased brain Mn levels. The PBPK model structure is comprised of compartments for the liver, lung, nasal cavity, bone, blood, cerebellum, olfactory bulb, globus pallidus, and pituitary gland with the remaining body tissues combined into a single compartment [77], to name a few. The PBPK model simulates concurrent exposure to dietary and inhaled Mn and also simulates ^54^Mn tracer kinetics from oral and inhalation exposure by intraperitoneal (ip), intravenous (iv), and subcutaneous (sc) administration. There has been improvement in the PBPK model since its development that used linear exchange rates to simulate Mn tissue kinetics under normal and deficient dietary conditions but lacked the sensitivity to detect the rapid increase in Mn tissue levels during inhalation exposure to high Mn concentrations [74]. Specifically, Andersen and colleagues have developed PBPK models that produced a consistent description of Mn tissue kinetics in monkeys and rats following dietary and inhalation exposures [78]. In addition, the previously established PBPK model structure for monkeys and rats [74] has been enhanced to exhibit fairly constant Mn levels during normal dietary intake and saturable Mn tissue stores that increased rapidly under inhalation resulting in an increased brain Mn concentrations in monkeys and rats [74]. The enhanced PBPK model structure has been extended to humans to predict inhalation exposure conditions that result in increased Mn concentrations via multiple exposure routes, including ip, iv, sc injection to simulate the distribution and elimination of the radioisotope ^54^Mn. The enhanced human PBPK simulation model structure recapitulates the biphasic elimination behavior for an exposure route and provides cross species descriptions of Mn tracer kinetics across multiple exposure routes. The globus pallidus Mn concentrations were unaffected by air concentrations < 10 µg/m^3^ Mn [77]. Parallel use of the human Mn PBPK simulation model and some of the aforementioned Mn detection and examination methods may be important for understanding human health risk assessments of Mn as it would require the consideration of various exposure routes, basal Mn tissue levels, and homeostatic control to explore conditions that may lead to tissue specific accumulation following chronic high- or low-level Mn overexposure.

## 6. Mn-Induced Parkinsonism and Parkinson’s Disease

Several metals are essential cofactors for enzymes and are required for optimal functioning of diverse cellular processes [9]. However, potential exposure to metals (both essential and non-essential) via multiple routes may cause detrimental effects in the brain or peripheral tissues [79,80]. Prolonged occupational exposure to metals (<20 years), including Mn, iron, mercury, zinc, aluminum, copper, and lead, may be a risk factor for PD [20,81]. Elevated intracellular levels of metals are known to induce deposition [82,83,84,85]. In 1837, Couper first described Mn-induced parkinsonism following his examination of Mn dioxide-exposed workers who worked in a Mn ore-crushing factory [86]. Excessive Mn accumulation in the basal ganglia of the brain, specifically in the globus pallidus, subthalamic nucleus, substantia nigra and striatum, which are involved in the control of motor and non-motor functions, causes progressive neuronal degeneration [87,88,89,90,91]. Mn toxicity is characterized by motor and sensory disturbances, as well as neuropsychiatric and cognitive deficits [92,93]. The motor impairments include hypertonia with cogwheel rigidity, bradykinesia, “cock-gait”, rapid postural tremor, and tendency to fall when walking backwards [15,94]. In human patients and animal models, neurons of the globus pallidus appear to be most sensitive to Mn-induced degeneration [95] while the striatum is less severely affected. Other brain areas that may be affected in Mn toxicity include the cerebellum, red nucleus, pons, cortex, thalamus, and anterior horn of the spinal cord [25]. This pathologic phenotype is distinct from idiopathic PD, where dopaminergic neurons of the substantia nigra pars compacta are specifically degraded [19]. Mn-induced parkinsonism is distinguishable from PD by the absence of Lewy bodies (another hallmark of PD) [19], the lack of therapeutic response to levodopa (a drug used to treat early stages of PD), failure to detect fluorodopa uptake by positron emission tomography (PET) studies, more frequent dystonia, and less resting tremor [96]. Patients occupationally exposed to excessive levels of Mn demonstrate a biphasic mode of physical decline comprising an initial phase of psychiatric abnormalities including emotional disturbance, memory loss, compulsive behavior, visual impairments, illusions and delusions, disorientation, and subsequent motor deficits such as bradykinesia, akinesia, and rigidity in the latter phase of the disorder [97,98]. Mn accumulation in the CNS is also associated with reproductive and developmental defects. For example, in one study, male workers occupationally exposed to Mn developed signs of parkinsonism, impotence and reduced libido [99,100]. Despite the pathologic and clinical distinctions between Mn-induced parkinsonism and PD, both disorders share generalized bradykinesia and widespread rigidity, as well as broadly similar pathophysiological mechanisms such as oxidative stress, protein aggregation, impaired proteasomal and autophagy functions, excitotoxicity, aberrant signal transduction, mitochondrial dysfunction and cell death pathways.

## 7. Mn-Induced Parkinsonism and the Involvement of the Dopaminergic System

Despite the evidence published thus far that links Mn neurotoxicity to dopaminergic dysfunction, there has been paucity, inconclusive, and contradictory evidence to explain the neurobiological basis for the effect of Mn on dopamine metabolism and neurotransmission, and selective accumulation of Mn in the basal ganglia region following overexposure. Dopamine mediates its regulatory actions via at least five receptors classified into two subtypes: D_1_-like (D_1_ and D_5_) and D_2_-like (D_2_, D_3_ and D_4_) receptors [101,102]. The D_1_-like and D_2_-like receptors interact with G-proteins that stimulate or inhibit adenylate cyclase activity respectively [102]. Several studies have reported a correlation between elevated Mn levels in the brain and dysregulated dopamine neurotransmission [103,104,105,106]. While some studies have reported no change in dopamine neurotransmission [107,108], other studies have reported increased [109,110], decreased [95,111,112,113], or both [114] in the tissue levels of dopamine levels post Mn intoxication. Enhanced Mn deposition in the basal ganglia is inversely related to dopamine levels both in adult [106] and neonatal [115] rats. Guilarte and colleagues utilized PET analysis and reported that chronic Mn exposure (7 weeks) causes a 36% decrease in striatal dopamine release compared to control group [116]. This may possibly be due to the inability of the radioligand to bind to the receptors as a result of endogenous dopamine displacement by the radioligand. Acute Mn exposure (1 month) in baboons caused a transient increase in DAT levels within a week of exposure that plateaued after 1 month [117]. In addition, *in vitro* Mn exposure altered dopamine transport kinetics (influx and efflux) to cause reduced dopamine uptake and amphetamine-induced dopamine efflux in DAT containing cells through enhanced trafficking of cell surface DAT into intracellular compartments [118]. Other studies have demonstrated a significant reduction in striatal D_2_ receptor levels following Mn exposure in the developing rats [106]. Neuroimaging techniques such as PET and SPECT used to examine nigrostriatal dopamine neuron terminal markers in workers occupationally exposed to Mn with Mn-induced movement abnormalities showed normal [^18^F]-fluorodopa PET, normal DAT SPECT and a decrease in D_2_ receptor levels [95,119,120]. Moreover, a summary of the effects of Mn-induced parkinsonism in humans by Huang showed a small but significant decrease in D_2_ receptor signals, which is opposite of what is observed in PD [119]. Indeed, a reduction in D_2_ function may be associated with an enhanced dopamine release and/or metabolism. Extracellular dopamine (approximately 80%) is recycled in the presynaptic neuron by the dopamine transporter (DAT) which is highly expressed in the caudate, putamen and nucleus accumbens, and increased throughout development to maximal levels in adulthood [121]. PET and SPECT analysis of nigrostriatal dopamine neuron terminal markers in PD patients showed a progressive loss of DAT and vesicular monoamine transporter 2 (VMAT2) and diminished 3,4-Dihydroxyphenylacetic acid (DOPAC) activity using [^18^F]-fluorodopa PET, as well as normal or increased levels of D_2_ receptor in the striatum [95,119,122,123]. Work on *Crassostrea virginica* (oysters) whose lateral gill cilia are controlled by the serotonergic-dopaminergic innervations from their ganglia showed that exposure to 100 µM Mn blocks the effects of dopamine on D_2_ type postsynaptic receptors [124]. These findings are consistent with the reported decrease in dopamine synthesis and dopamine transporter (DAT) levels in PD patients [125]. The summary by Huang has provided evidence for the absence of nigrostriatal dopamine neuron degeneration in occupationally Mn-induced parkinsonism [95]. Kim and colleagues have challenged the axiom and over simplification of the idea that Mn causes parkinsonism by pallidal degeneration. The authors examined two patients (48 and 56 years old men) with a clinical history of occupational Mn exposure presented with Mn-induced parkinsonism and fifteen healthy volunteers (six men and nine women) aged matched in clinical studies. The 48-year old patient showed symmetrical high MRI signal intensities in the globus pallidus and substantia nigra on T1-weighted images and increased Mn concentrations in the blood and urine, which are consistent with Mn exposure. However, the patient’s clinical features were typical of idiopathic PD, The use of [^123^I]-(1r)-2β-carboxymethoxy-3β-(4-iodophenyl)tropane ([^123^I]-b-CIT) that is known to bind to DAT with high affinity and low nonspecific binding [126] in single-photon emission computed tomography showed severe reduction of striatal β-CIT binding in the two patients with Mn-induced parkinsonism, which is consistent with PD. The above results led the authors to propose three interpretations: (i) the patients have idiopathic PD, and Mn exposure is incidental; (2) Mn induces selective degeneration of presynaptic dopaminergic terminals, thereby causing parkinsonism; (3) Mn exposure acts as a risk of idiopathic PD in the two patients [127]. Due to the small sample size in Kim’s study, further research in humans with similar Mn exposure levels and clinical symptoms would be required to elucidate the above proposed interpretations. Overall, the bulk of the evidence suggests that Mn functionally alters neurotransmission through the dopaminergic system without altering the morphology of dopaminergic neurons suggesting that there are fundamental differences in the changes induced in the dopaminergic system during Mn toxicity and idiopathic PD. Additionally, other monoaminergic neurotransmitters such as serotonin, epinephrine and norepinerphrine are also impaired in Mn-induced parkinsonism. For example, non-human primates intoxicated with Mn exhibit diminished tissue levels of serotonin and norepinephrine [20,128]. These studies suggest that Mn alters the integrity of monoaminergic neurotransmission.

## 8. Mn-Induced Parkinsonism and GABA System

Mn accumulates primarily in the globus pallidal GABAergic neurons of the basal ganglia. The effects of Mn on GABAergic neurotransmission are controversial. For example, previous studies have reported conflicting results including (i) no statistical difference in brain GABA levels in intravenously exposed primates [129] (ii) no effect of Mn oxide on GABA_a_ receptor levels in nonhuman primates after monthly injections for 2 years [130] (iii) marginal trend (*p* < 0.1) towards diminished pallidal GABA levels in monkeys following MnSO_4_ exposure [128] (iv) decreased GABA levels in the brain after dietary treatment of rats with 10 ppm Mn [104] and (v) significantly elevated striatal GABA levels in rats following Mn exposure [131,132,133,134,135,136]. In recent studies, Anderson and colleagues have demonstrated that Mn exposure caused a decrease in brain GABA levels and uptake in synaptosomes [137]. A recent study of ten Mn-exposed smelters and ten matched controls used the MEGA-PRESS-IVS method that combines MEGA (a frequency-selective editing technique) editing with the point-resolved spectroscopy sequence (PRESS) and inner volume saturation (IVS) localization that reduces the signal-to-noise ratio (SNR) to determine GABA concentrations [138] in several brain regions including the frontal cortex, globus pallidus, thalamus, and putamen. The authors reported increased GABA levels by 80% on average in the thalamus and adjacent brain regions in the Mn-exposed group, which corroborated the other rodent studies mentioned above. In addition, a 3D high-resolution T1-weighted MRI sequence was used to investigate differences in Mn deposition in the brain as determined by pallidal index (PI), a ratio of the T1-weighted signal intensities referenced to a white matter region in the brain with no to little Mn deposition. The authors reported a group difference between exposed and non-exposed subjects using the PI (*p* = 0.007) with only seven out of the ten exposed subjects that showed hyper-intense signal in the T1-weighted images revealing Mn deposition [139]. Since the MRI signal hypersensitivity that is indicative of high Mn deposition in the brain did not correlate with the GABA levels, it is plausible that Mn neurotoxicity may be defined by the intrinsic vulnerability of neuronal systems to injury rather than the net accumulated tissue Mn levels as previously proposed by Burton and Guillarte, 2009 [129]. It is also possible that the conflicting effects of Mn on GABA homeostasis are due to diverse experimental techniques used to examine the effect of extracellular GABA homeostasis and transport dynamics. More research is necessary to establish the effect of Mn exposure on GABA neurotransmission.

## 9. Mn-Induced Parkinsonism and Glutamate System

As previously noted, Mn is an essential metal cofactor for the abundant manganoprotein glutamine synthetase [GS] predominantly expressed in astrocytes. Glutamine synthetase synthesizes glutamine via the conversion of glutamate to glutamine. Importantly, Mn has been suggested to regulate glutamine synthetase activity. Reduced GS activity in the brain has been proposed to increase glutamate trafficking and glutamatergic signaling, which results in excitotoxicity (excessive amounts of glutamate at the synapse) [140]. Eighty percent of synaptic glutamate is cleared by astrocytes via the glutamate:aspartate transporter (GLAST) also referred to as excitatory amino acid transporter (EAAT) [141,142]. Recognizing the essentiality of the intricate regulation of Mn homeostasis at the glutamatergic synapse, it is not surprising that Mn deposition in the basal ganglia has been reported to alter glutamatergic neurotransmission. Immunohistochemical analysis in *Cynomolgus macaques* exposed to Mn showed reduced GS in the globus pallidus, which suggests the possible dysfunction in the synaptic and/or astrocytic glutamate neurotransmission [129]. Exposure to Mn reduced GLAST [108] expression and glutamate uptake in astrocytes that may underlie the increased extracellular glutamate levels at the synapse [7,104]. Excess Mn deposition in the basal ganglia showed enhanced sensitivity of postsynaptic glutamate receptors via stimulation of glutamate receptors [143]. This response is suggested to cause abnormal activation of pallidal neurons that causes motor dysfunction [8]. On the other hand, chronic Mn intoxication causes hyperactivity of corticostriatal neurons and subsequently enhances the frequency and amplitude of spontaneous excitatory postsynaptic potentials (EPSPs) in the striatum [144]. Moreover, chronic Mn exposure alters glutamatergic neurotransmission through constitutive activation of ionotropic glutamate receptors, altered synaptic ionic concentration and homeostasis, mitochondrial dysfunction [7,145,146,147,148] and impaired cellular metabolism [147,148]. Mn intoxication causes elevated synaptic glutamate levels and reduced glycine interaction with N-methyl-d-aspartate (NMDA) subtype of glutamate receptors in mouse brains [149]. Burton and colleagues have reported no change in NMDA receptor expression following Mn intoxication in nonhuman primates [129]. The exact mechanism underlying how deposited Mn in the basal ganglia modulates glutamatergic neurotransmission remains contradictory. However, Fitsanakis and colleagues have postulated that accumulated Mn in the brain may decrease astrocytic clearance of glutamate from the synapse and enhance sensitivity of glutamate receptors, which causes mitochondrial dysfunction, oxidative stress and eventual neuronal loss [8].

## 10. Preventive and Treatment Strategies for Mn-Induced Parkinsonism and PD

Due to the fact that Mn-induced neurotoxicity is mainly through occupational hazards, it is important to develop workplace regulation and to educate individuals potentially exposed to Mn via welding or mining on the strategies to minimize and/or avoid overexposure to Mn. For example, the Occupational Safety and Health Administration (OSHA) sets standards for Permissible Exposure Levels (PEL) and in its hazard communication standard requires employers to provide information and training for workers on hazardous materials in the workplace. Proper ventilation and exhaust systems and the substitution of a lower fume-generator or less toxic welding type or consumable are recommended [150]. Since the introduction of levodopa in 1968 for the treatment of PD, studies have reported a duration and age of treatment-dependent complications such as dyskinesia, weaning off, and “on-off” phenomenon [151,152,153,154] in ~100% patients with early onset PD, ~80% of levodopa-treated patients for 10 years, and ~50% of patients who received levodopa treated for at least 5 years [155,156,157]. Although levodopa treatment induces continuous dopaminergic stimulation to ameliorate the dopamine deficiency, emerging evidence suggests that it may also target other neuronal populations that express acetylcholine and glutamate to achieve its role [156,158,159]. Mn-induced parkinsonism patients regardless of the dose of levodopa administered are unresponsive to levodopa treatment [62,160,161] possibly due to the relatively intact nigrostriatal pathway in the latter phase of the disorder [25]. It is noteworthy that Mn catalyzes dopamine autooxidation to produce toxic quinones and semiquinones that contraindicates levodopa treatment in Mn-induced parkinsonism [162,163]. Overall, current evidence suggests that the lack of responsiveness to levodopa is a hallmark of Mn-induced parkinsonism, which distinguishes the disease from idiopathic PD.

Despite the paucity of large clinical trials for chelation therapy in manganism, studies have demonstrated reversal and clinical improvement in symptoms amongst Mn-induced parkinsonism patients and animal models using chelation treatment [164,165,166,167,168,169]. Chelators have also been successfully used to alleviate parkinsonian symptoms in some patients with familial Mn-induced parkinsonism due to SLC30A10 mutations [58,59]. Ethlyene diamine tetraacetic acid (EDTA) is a chelator used commercially as a metal sequester in food additives [25]. Proper use of its calcium disodium salt (CaNa_2_EDTA) by trained personnel on a hospital basis and careful examination of the patients, as well as cautious management of the administration dosage, schedule and procedures have demonstrated protection of renal function and tissue integrity in Mn-induced parkinsonism [170]. Furthermore, CaNa_2_EDTA treatment reduced Mn-induced dopamine autooxidation *in vitro* [171], enhanced urinary excretion of Mn in humans [172] and reduced Mn levels in the brain and liver of Mn-exposed rats [173]. Nevertheless, other studies involving Mn-induced parkinsonism patients with extremely high occupational exposures have reported lack of clinical amelioration [174,175,176] or relinquished clinical amelioration [177] upon CaNa_2_EDTA treatment. Likewise, para-aminosalicylic acid (PAS), an FDA-approved anti-tuberculosis drug, has been used successfully in the treatment of severe Mn poisoning in a patient exposed to airborne Mn for 21 years [165]. Recent studies aimed at understanding the efficacy of PAS in attenuating Mn intoxication in rats showed that PAS treatment reduced Mn accumulation, neuroinflammation, oxidative stress and locomotor activity impairments in rats exposed to Mn [178]. Furthermore, an *in vitro* study has showed PAS inhibition and hippocampal injury after Mn exposure [179]. These and other findings elucidate the importance of chelation therapy in manganism and provide the basis for the development, clinical trial conformation and establishment of alternative efficacious therapies for manganism.

In general, diagnosis of PD relies on the clinical presentation of four cardinal motor signs, tremor, bradykinesia, rigidity and postural instability [180]. While dopamine replacement therapy improves tremor, rigidity and bradykinesia in individuals with PD, its effect on postural instability is limited suggesting that postural instability is a dopamine resistant motor deficit [181,182,183]. Further, the non-motor symptoms including dysfunctions in autonomic, cognitive, and psychiatric observed in PD are poorly responsive, irresponsive or exacerbated by anti-parkinsonism medications including levodopa [184]. In spite of the recent advances in drug discovery that have provided multiple strategies to alleviate some symptoms and decrease progression, there is yet no cure for PD. Chiefly, the use of monoamine oxidase-B (MAO-B) inhibitors such as selegiline and rasagiline that prevents the breakdown of dopamine in PD patients attenuates parkinsonism and delays the need for levodopa treatment by several months [185,186]. Anticholinergic drugs such as amantadine, benztropine, biperiden, and procyclidene known to be the oldest and cheapest anti-parkinsonism drugs have been useful adjuvants to levodopa treatment that effectively ameliorates tremor and rigidity without alterations in bradykinesia [180]. Furthermore, surgical procedures such as high frequency stimulation of the internal capsule of the globus pallidus (GPi) and subthalamic nucleus (STN) are used to treat PD patients with severe clinical presentation that are poorly responsive to the aforementioned drug strategies [187,188]. Finally, gene therapy aimed at repairing affected dopaminergic neurons via packaging and delivering of deficient enzymatic machinery, as well as the use of nanotechnology for packaging small drugs or nanoparticles to enable readily passage across the blood brain barrier are currently being developed for treatment of PD.

## 11. Conclusions

While Mn-induced parkinsonism and PD share some similarities in their pathophysiological mechanisms and few motor symptoms, there are striking differences in the clinical and pathologic manifestations between both disorders. Future work aimed at understanding the basis for the selective neuropathology of Mn-induced parkinsonism and PD will be necessary to better understand the biology of these two related yet separate diseases and for the development of drugs to treat both disorders. In addition, considerations of the pharmacokinetics of Mn and its concurrent effects on multiple neurotransmission systems will be beneficial. Finally, more research on the influence of aging, gender and epigenetics in Mn-induced parkinsonism, as well as the synergism between Mn neurotoxicity and PD risk genes may possibly contribute to the advancement of drug discovery for these disorders.
